# Slowing of Hippocampal Activity Correlates with Cognitive Decline in Early Onset Alzheimer’s Disease. An MEG Study with Virtual Electrodes

**DOI:** 10.3389/fnhum.2016.00238

**Published:** 2016-05-20

**Authors:** Marjolein M. A. Engels, Arjan Hillebrand, Wiesje M. van der Flier, Cornelis J. Stam, Philip Scheltens, Elisabeth C. W. van Straaten

**Affiliations:** ^1^Alzheimer Center and Department of Neurology, Neuroscience Campus Amsterdam, VU University Medical CenterAmsterdam, Netherlands; ^2^Department of Clinical Neurophysiology and Magnetoencephalography Center, Neuroscience Campus Amsterdam, VU University Medical CenterAmsterdam, Netherlands; ^3^Department of Epidemiology and Biostatistics, Neuroscience Campus Amsterdam, VU University Medical CenterAmsterdam, Netherlands; ^4^Nutricia Advanced Medical Nutrition, Nutricia ResearchUtrecht, Netherlands

**Keywords:** Alzheimer’s disease, MEG, source-space, beamformer, virtual electrode, hippocampus, relative power, peak frequency

## Abstract

Pathology in Alzheimer’s disease (AD) starts in the entorhinal cortex and hippocampus. Because of their deep location, activity from these areas is difficult to record with conventional electro- or magnetoencephalography (EEG/MEG). The purpose of this study was to explore hippocampal activity in AD patients and healthy controls using “virtual MEG electrodes”. We used resting-state MEG recordings from 27 early onset AD patients [age 60.6 ± 5.4, 12 females, mini-mental state examination (MMSE) range: 19–28] and 26 cognitively healthy age- and gender-matched controls (age 61.8 ± 5.5, 14 females). Activity was reconstructed using beamformer-based virtual electrodes for 78 cortical regions and 6 hippocampal regions. Group differences in peak frequency and relative power in six frequency bands were identified using permutation testing. For the patients, spearman correlations between the MMSE scores and peak frequency or relative power were calculated. Moreover, receiver operator characteristic curves were plotted to estimate the diagnostic accuracy. We found a lower hippocampal peak frequency in AD compared to controls, which, in the patients, correlated positively with MMSE [*r*(25) = 0.61; *p* < 0.01] whereas hippocampal relative theta power correlated negatively with MMSE [*r*(25) = -0.54; *p* < 0.01]. Cortical peak frequency was also lower in AD in association areas. Furthermore, cortical peak frequency correlated positively with MMSE [*r*(25) = 0.43; *p* < 0.05]. In line with this finding, relative theta power was higher in AD across the cortex, and relative alpha and beta power was lower in more circumscribed areas. The average cortical relative theta power was the best discriminator between AD and controls (sensitivity 82%; specificity 81%). Using beamformer-based virtual electrodes, we were able to detect hippocampal activity in AD. In AD, this hippocampal activity is slowed, and correlates better with cognition than the (slowed) activity in cortical areas. On the other hand, the average cortical relative power in the theta band was shown to be the best diagnostic discriminator. We postulate that this novel approach using virtual electrodes can be used in future research to quantify functional interactions between the hippocampi and cortical areas.

## Introduction

Alzheimer’s disease (AD) is a neurodegenerative disease affecting a large proportion of the human population worldwide. While the prevalence of AD increases with advancing age, an early onset form of the disease is increasingly recognized (van der Flier and Scheltens, 2005). Early onset AD is frequently referred to as a disease onset before the age of 65, although this cutoff age is arbitrary (van der Flier et al., 2011). Electroencephalography (EEG) studies have been conducted for several decades and have consistently demonstrated slowing of oscillatory brain activity in AD (Jeong, 2004; de Waal et al., 2012). This slowing of oscillatory frequencies in AD may be caused by the loss of connection between neurons, shown in a model of coupled neural masses (de Haan et al., 2012; Stam and van Straaten, 2012). These losses of connections on a cellular level are also observed at the macroscopic level using functional connectivity. This resulted in hypotheses of AD as a disconnection syndrome (Delbeuck et al., 2007). Studies with magnetoencephalography (MEG) have consistently reproduced the slowing of brain activity in AD (e.g., Fernández et al., 2006b; de Haan et al., 2008; Abatzoglou et al., 2009). The relation between slowing of oscillatory brain activity and decreasing connectivity has been modeled using computer based neuronal assemblies while it has been shown that amyloid-beta toxicity primarily affects dendrites (Thal et al., 2008). Studies, in which slowing of oscillatory brain activity has been reported using MEG, were all conducted in “signal space” meaning that an accurate regional estimation is lacking. MEG measures neuronal activity directly with a high temporal and spatial resolution (Ioannides, 2006; Stam, 2010; van Straaten et al., 2014), allowing accurate source reconstruction (Baillet et al., 2001; Hillebrand and Barnes, 2005). Techniques to transform signal space data to “source space” data are not only able to localize MEG signals that arise from the cortex, but neuronal activity in subcortical gray matter, including the medial temporal lobe, can also be localized (Attal and Schwartz, 2013). This allows researchers to non-invasively look at specific brain regions, not only cortically but also subcortically. A major region of interest in AD would be the hippocampus, since this region is already involved in the early stages of the disease.

The purpose of this study was to non-invasively characterize resting-state brain activity from deeper brain structures, namely the hippocampi, and to compare this to cortical activity and cognitive performance. Furthermore, we aimed to compare differences in hippocampal and cortical activity between AD patients and healthy controls, where we expected a slowing in the patients compared to the healthy controls. Our results show that the use of beamformer-based virtual electrodes indeed allow for the non-invasive sampling of these deeper brain structures, as we characterized, for the first time, resting-state brain activity in AD in the hippocampus, as well as in cortical regions.

## Materials and Methods

### Subjects

We conducted MEG measurements in 27 young (age: 60.6 ± 5.4 years) patients with probable AD from the Amsterdam Dementia Cohort in the Alzheimer Center of the VU University Medical Center. All patients fulfilled the National Institute of Aging-Alzheimer’s Association (NIA-AA) criteria for probable AD with a high likelihood of AD pathophysiology, based on the combination of a positive biomarker reflecting Aβ deposition (in either cerebrospinal fluid (CSF) or by positron emission tomography (PET) scanning) and/or a positive biomarker for neuronal injury (tau or phosphorylated tau in CSF). They were assessed according to a standard diagnostic workup for dementia screening including an informant-based history of the patient (if available), physical-, neurological and cognitive examinations, laboratory tests, structural brain imaging, and EEG. Diagnoses were made in a multidisciplinary consensus meeting. None of the patients had a known autosomal dominant mutation. Patients gave written informed consent for use of their clinical data for research purposes (van der Flier et al., 2014). For our analyses, we used the following clinical data: subject characteristics, mini-mental state examination (MMSE). Exclusion criteria for participation were: an active psychiatric or neurologic disorder, MMSE-score below 18, or age above 70 years. In addition to the patient group, we included 26 of 31 non-demented controls that responded to an advertisement in a national newspaper. After a telephone interview to exclude neurologic or psychiatric disorders, subjects underwent neuropsychological testing, magnetic resonance imaging (MRI) of the brain and an MEG recording. All MEG recordings were obtained one to several hours before, or more than 1 week after, the MRI-scan in order to avoid interference due to, for example, magnetized dental elements. One volunteer was excluded as a meningioma found on the MRI; four volunteers were excluded due to poor performance during neuropsychological testing. The local Ethics Committee approved the study and all participants gave written informed consent before participation.

### MEG Recordings

Magnetoencephalography recordings were obtained within several weeks after diagnosis. In order not to interfere with the resting-state condition, neuropsychological testing of the healthy control subjects was conducted after the MEG-recording. Since patients received an AD diagnosis after they went to the Alzheimer’s center, the MEG took place a few weeks after the MRI scan and neuropsychological testing. All MEG recordings were made in a magnetically shielded room (VacuumSchmelze GmbH, Hanua, Germany) using a 306-channel whole-head system (Elekta Neuromag Oy, Helsinki, Finland). The recording protocol consisted of 5 min of eyes-closed resting-state condition followed by 2 min-eyes open, and again 5 min eyes-closed. Only data from the second eyes-closed session were analyzed here. The recordings were sampled at 1250 Hz, with an online anti-aliasing filter (410 Hz) and high-pass filter (0.1 Hz). Oﬄine, a spatial filter, the temporal extension of Signal Space Separation (tSSS; Taulu and Simola, 2006; Taulu and Hari, 2009), as implemented in MaxFilter software (Elekta Neuromag Oy, version 2.2.10), was applied with a sliding window of 10 s. Channels containing excessive artifacts were manually discarded after visual inspection of the data by one of the authors (ME) before estimation of the SSS coefficients. The number of excluded channels varied between 1 and 12. After fine-tuning for acquisition conditions at our site, the tSSS filter was used to remove noise signals that SSS failed to discard, typically from noise sources near the head, using a subspace correlation limit of 0.9 (Medvedovsky et al., 2009). Typical artifacts were due to (eye) movements, swallowing, dental prosthetics, or drowsiness, although the subjects were instructed to stay awake and reduce eye movements during the MEG recording. The head position relative to the MEG sensors was recorded continuously using the signals from four head-localization coils. The head-localization coil positions were digitized, as well as the outline of the participant’s scalp (∼500 points), using a 3D digitizer (FasTrak, Polhemus, Colchester, VT, USA). This scalp surface was used for co-registration with the patients’ structural scan.

### Co-registration of MEG Data and Structural Scans

Structural MRI scans were made of all participants. For one AD patient, a computer tomography (CT) scan was used instead of an MRI because of insufficient quality of the MRI. For all participants, the outline of the scalp on the structural scans was extracted. Co-registration of the MEG data with the structural scans was achieved using surface matching, resulting in an estimated co-registration accuracy of approximately 4 mm (Whalen et al., 2008). Visual inspection of the co-registration between the MEG- and the MRI/CT scalp surfaces was performed for all patients.

### Source Reconstruction in Cortical Regions and Hippocampi Using Virtual Electrodes

In order to obtain source localized activity in cortical regions, we applied an atlas-based beamformer approach (Hillebrand et al., 2012). In this work, we used the sphere that best fitted the scalp surface [using the Nelder–Mead method (fminsearch in Matlab, version R2008b)] as a volume conductor model. Sensor signals are projected to an anatomical framework such that source-reconstructed neuronal activity for 78 cortical regions-of-interest (ROIs; Gong et al., 2009), identified by means of automated anatomical labeling (AAL; Tzourio-Mazoyer et al., 2002; Supplementary Table S1), is obtained. The voxel with the maximum pseudoZ (Hillebrand et al., 2012) in a particular frequency band (see below) was selected as representative for that specific ROI (Zobay et al., 2015). Once the broad band (0.5–48 Hz) beamformer weights for the selected voxel were computed, then the time series for this voxel, i.e., a virtual electrode, was reconstructed (see Hillebrand et al., 2012 for details).

**FIGURE 1:**
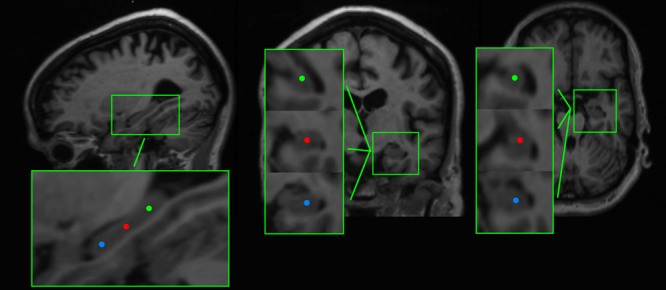
**Example of the placement of virtual electrodes in one patient in the right hippocampus on T1 weighted magnetic resonance imaging (MRI).** The virtual electrodes are shown as blue (anterior), red (center), and green (posterior) dots on sagittal, coronal and axial brain slices (left, middle, and right panel, respectively).

To reconstruct brain activity for the hippocampus, three virtual electrodes were manually placed in both left and right hippocampal gray matter by one of the authors (ME). The first electrode was placed in the center of the hippocampi while the second and the third electrode were placed anterior and posterior from the central electrode, respectively (**Figure 1**). The center of the hippocampus, in which the central virtual electrode was placed, was identified on the sagittal MRI coupe in which the full length of the hippocampus was visible. Thereafter, the posterior and anterior virtual electrodes were placed with an approximately equal distance (dependent on the size of the hippocampus) to the centrally placed virtual electrode in the same coupe, leading to electrode placements within the hippocampal gray matter. Virtual electrodes could not be reliably placed in 4 out of 27 AD patients (one patient with CT, and four patients with MRI of insufficient quality). Therefore, we placed hippocampal virtual electrodes in 23 AD patients and 26 healthy controls (**Table 1**).

**Table 1:** Subject characteristics.

	AD patients		Healthy controls
	Cortical analyses	Hippocampal analyses	Cortical- and hippocampal analyses
*N*	27	23	26
Mean age (SD)	60.6 (5.4)	60.7 (5.6)	61.8 (5.5)
Gender (F/M)	12/15	11/12	14/12
Mean MMSE (SD)	23.4 (2.6)^∗∗^	23.2 (2.5)^∗∗^	28.9 (1.0)
Mean educational score (SD) ^a^	4.84 (1.06)	4.70 (0.93)	5.71 (0.91)
Presence of dementia in family (yes/no/unknown)^b^	10/15/2	9/12/2	N.A.
APOE genotype	E4E4: 10	E4E4: 9	N.A.
	E3E4: 13	E3E4: 10	
	E3E3: 2	E3E3: 2	
	E2E4: 1	E2E4: 1	

*APOE, apolipoprotein E alleles; MMSE, mini-mental state examination; F, number of female subjects; M, number of male subjects; N, number of subjects; N.A., not available; SD, standard deviation. ^∗∗^*p* < 0.01; ^a^level of education was rated according to Verhage (Verhage, 1964); ^b^positive history indicating at least one first degree relative with dementia.*

For each subject, care was taken to select 20 artifact-free epochs of 4096 samples (3.2768 s) by one of the authors (ME). A second researcher independently evaluated the selected epochs. Epochs without consensus were replaced by new epochs. Epochs were converted to ASCII-files and imported into an in-house developed software package (BrainWave version 0.9.125, CS. Software^1^). The MEG data were digitally filtered with a band pass filter of 0.5–48 Hz using a fast Fourier transform, following which the relative power, averaged over the selected epochs, was estimated for the following frequency bands: delta (0.5–4 Hz), theta (4–8 Hz), lower alpha (8–10 Hz), upper alpha (10–13 Hz), beta (13–30 Hz), and gamma (30–45 Hz). All real and imaginary components of the Fourier transform outside the pass band were set to 0, following which an inverse Fourier transform was used to obtain the filtered time series. Average peak frequency values were obtained by averaging, over epochs, the peak frequency within the 4–13 Hz frequency range.

### Statistical Analysis

IBM SPSS Statistics 20.0 for mac and R 3.2.0 for mac were used for statistical analyses. First, we tested subject characteristics using an unpaired student’s *t*-test or χ^2^ test where appropriate. We calculated relative power values for each cortical ROI and for all six hippocampal virtual electrodes. We compared AD and controls by means of permutation analysis (Nichols and Holmes, 2002) in a similar way as described by Ponsen et al. (2012). This approach has been reported to show robust results in MEG data (Singh et al., 2003). Hereby, a null distribution for between-group differences (independent *t*-test) was derived by permuting group assignment and calculating a *t*-statistic after each permutation. To correct for multiple comparisons, the maximum *t*-value across ROIs of each permutation was used to construct a distribution of maximum *t*-values (*N* = 1000) against which the observed *t*-values were tested (with α = 0.05). Receiver operating characteristic (ROC) curves were plotted for the peak frequency and the power in the different frequency bands for both cortical areas (averaged over all 78 cortical regions) and the hippocampi (averaged over the six hippocampal regions). The optimal thresholds of the cut-offs were determined using the Youden-index. Bivariate non-parametric correlation (Spearman) tests of average relative power or average peak frequency with MMSE scores were performed for both the cortical areas and the hippocampi. Correlations were estimated only for the patients.

## Results

### Subject Characteristics

Subject characteristics are given in **Table 1**. Continuous and categorical variables were tested by unpaired Student’s *t* and χ^2^ tests, respectively. Age, gender, and level of education did not differ between groups. The mean MMSE score was lower in AD patients compared to the healthy controls.

### Hippocampal Peak Frequency

**FIGURE 2:**
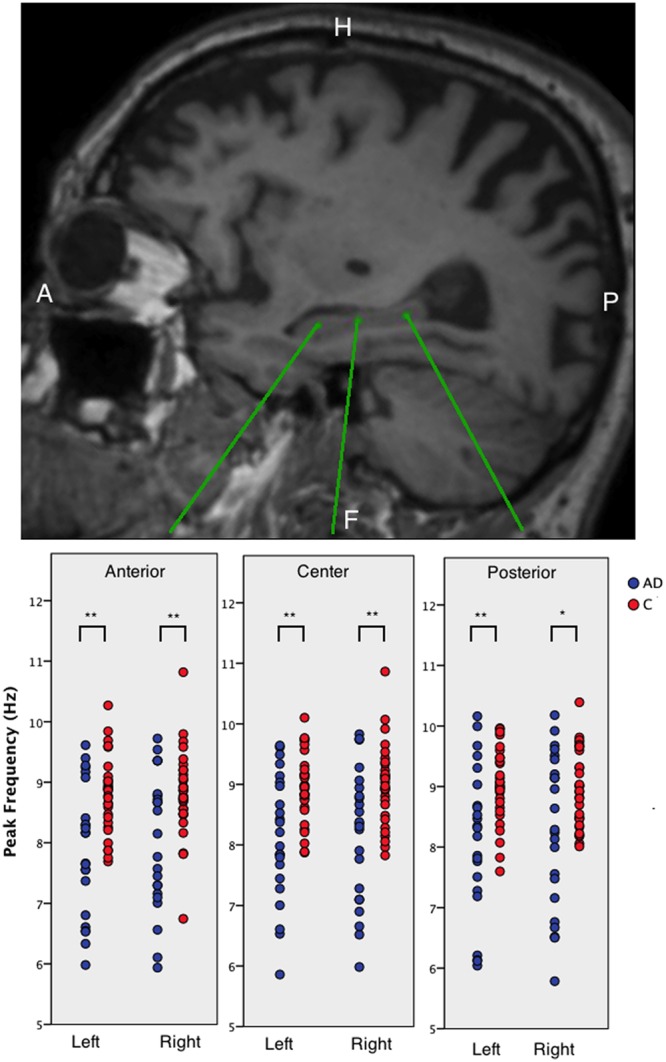
**The peak frequency for virtual electrodes placed in the hippocampi.** Top panel shows an example sagittal view of a T1-weighted MRI with three different virtual electrode in one patient in the left hippocampus. The lower panel shows boxplots of the anterior, middle, and posterior left and right hippocampal regions for AD patients and healthy controls. Significant differences are indicated using asterisks: ^∗^*p* < 0.05, ^∗∗^*p* < 0.01.

We found that AD patients had significantly lower peak frequencies compared to controls (C) in all six regions [left anterior AD (mean ± standard deviation): 7.91 ± 1.09, *C*: 8.77 ± 0.63; left center AD: 8.14 ± 1.04, *C*: 8.94 ± 0.57; left posterior AD: 8.12 ± 1.23, *C*: 9.01 ± 0.61; and right posterior AD: 8.27 ± 1.27, *C*: 9.01 ± 0.61; right anterior AD: 8.02 ± 1.12, *C*: 8.82 ± 0.72; right center AD: 8.15 ± 1.09, *C*: 8.98 ± 0.66; all regions: *p* < 0.01; except right posterior *p* < 0.05]. We found no differences in peak frequency between the three hippocampal areas (anterior, center, and posterior hippocampus) or between the left and right hippocampi (**Figure 2**).

### Hippocampal Relative Power

Since location within the hippocampus was not relevant, we averaged the relative power values over sub-regions and for the left and right hippocampi for every subject. We found higher relative theta power in AD patients compared to controls (AD: 0.19 ± 0.06, *C*: 0.14 ± 0.03, *p* < 0.01). Upper alpha and beta band relative power was lower in AD patients compared to controls (upper alpha band AD: 0.11 ± 0.03, *C*: 0.13 ± 0.03; beta band AD: 0.21 ± 0.07, *C*: 0.25 ± 0.04; all *p* < 0.01).

### Cortical Peak Frequency

**FIGURE 3:**
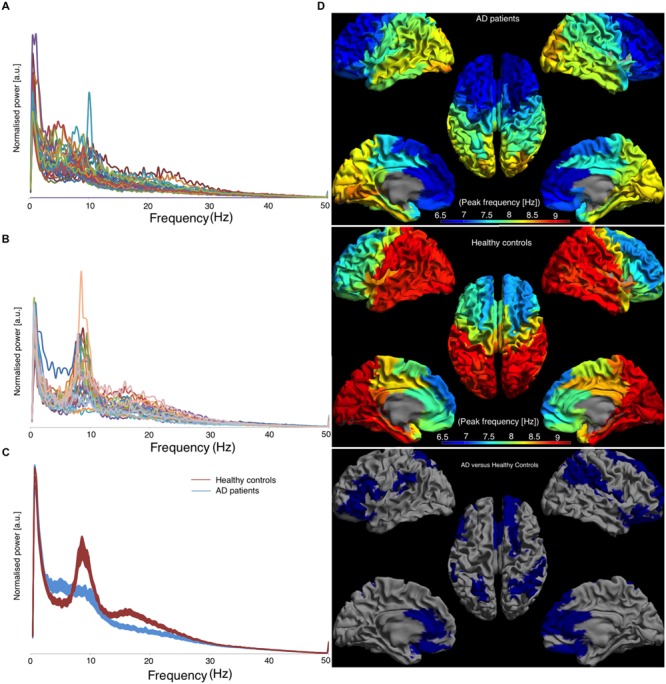
**Left panel shows the frequency spectra, averaged over all cortical ROIs.** [a.u.] stands for arbitrary units, **(A)** represents AD patients, **(B)** represents healthy controls, and **(C)** represents the average spectra for both groups with the corresponding standard errors and the right panel shows the regional peak frequency for each ROI **(D)**. **(D)** A color-coded map on a template mesh of the peak frequency values for every region of interest. The same color scale is used for the AD patients and the healthy controls. The lower part of panel **(D)** shows the significant differences (blue means AD patients have lower values than healthy controls) between the two groups as determined using permutation analyses (*p* < 0.05, corrected).

Individual power spectra, averaged over all 78 cortical ROIs, for AD patients and healthy controls are presented in **Figures 3A,B**, respectively. Group averages of the spectra, including the standard error of the mean, are in **Figure 3C**. The mean peak frequency, averaged over all ROIs, was lower for the AD patients than for the controls (AD 7.63 ± 0.98; controls 8.49 ± 0.53; *p* < 0.001). Regionally, we found decreased peak frequencies in AD patients compared to controls (**Figure 3D**) in 26 regions (Supplementary Table S2) using permutation testing. Note that mainly association cortical areas showed a lower peak frequency in AD patients compared to healthy controls, whereas the peak frequencies for the primary cortical areas were not different between the groups (Supplementary Table S2).

### Cortical Relative Power

**FIGURE 4:**
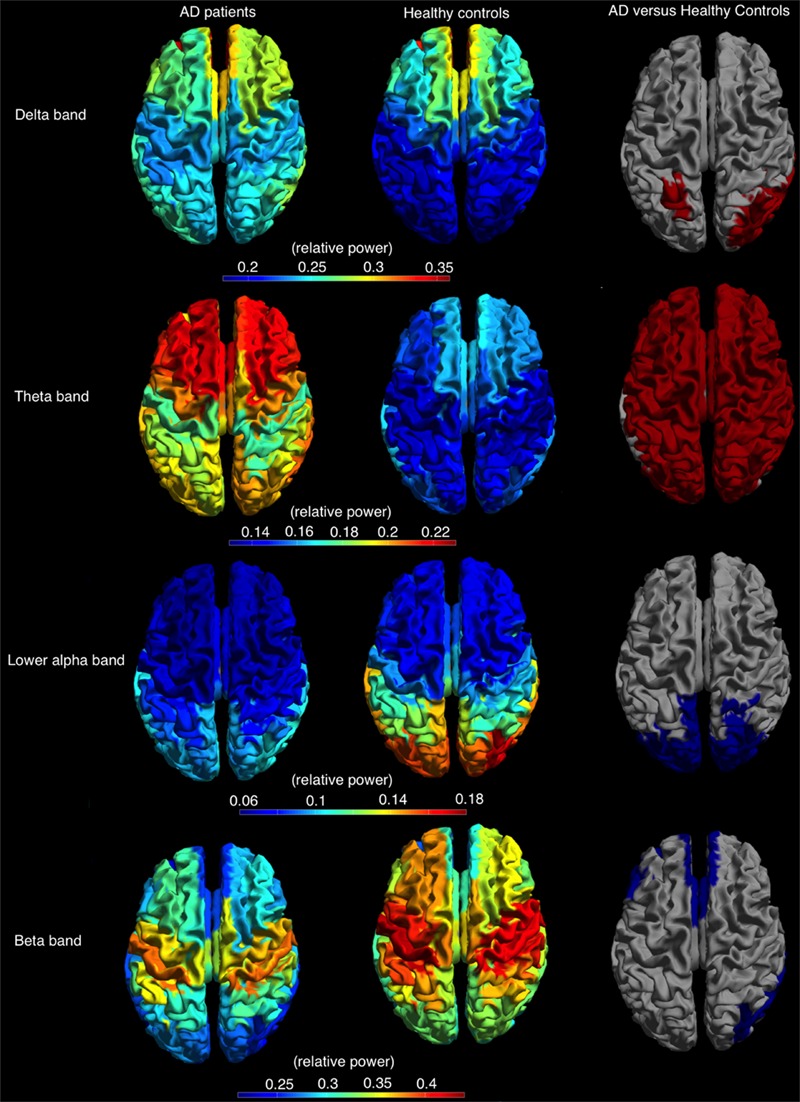
**Relative power, shown as a color-coded map on a template mesh, for the AD patients **(left)** and healthy controls **(middle)** in, respectively, the delta (first row), theta (second row), lower alpha (third row), and beta (fourth row) bands.** The **(right)** shows the significant differences (red and blue indicate higher and lower relative power values for AD patients compared to healthy controls, respectively) in corresponding bands between the two groups as determined using permutation analysis (*p* < 0.05, corrected).

The relative power, averaged over all cortical regions, was lower in AD patients compared to controls in lower alpha and beta band (lower alpha band: AD 0.09 ± 0.02, *C*: 0.11 ± 0.04, *p* < 0.05; beta band: AD 0.27 ± 0.06, *C*: 0.32 ± 0.05, *p* < 0.05) and higher in the delta and theta band (delta band: AD 0.27 ± 0.05, *C*: 0.23 ± 0.05, *p* < 0.05; theta band: AD 0.20 ± 0.05, *C*: 0.15 ± 0.03, *p* < 0.05). Regional differences in relative power using permutation testing are presented in **Figure 4** (only bands with at least one region that showed a significant difference are presented) and Supplementary Table S3. The delta power was higher in AD than in controls in six regions, all located caudally from the central sulcus (left superior parietal gyrus, right angular gyrus, right superior, middle and inferior occipital gyrus, and right middle temporal gyrus). The theta band power was higher in almost all cortical regions in AD versus controls. The power in the lower alpha band was lower in AD versus controls in 16 regions, all in the parietal/occipital lobe, whereas the power in the beta band was lower in 17, mostly frontal, regions (**Figure 4**).

### Diagnostic Accuracy

**FIGURE 5:**
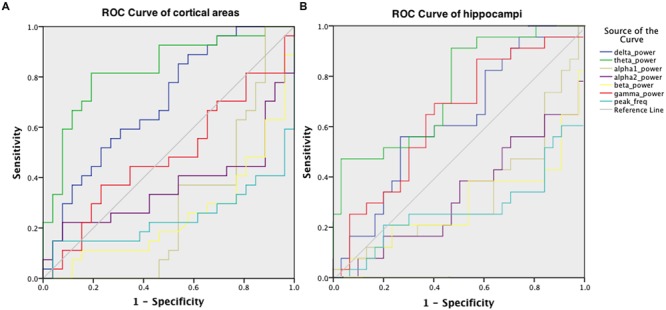
**ROC for discriminating AD patients and healthy controls, obtained using the relative power values in six frequency bands and the peak frequency, averaged over cortical areas **(A)** and hippocampal areas (B)**.

Receiver operator characteristic curves were plotted to investigate the discriminative capability of the relative power and peak frequency both using the averaged values over the cortical (**Figure 5A**) and hippocampal (**Figure 5B**) areas for the various frequency bands. The best diagnostic accuracy was observed for the cortical relative theta power (area-under-the-curve of 0.830) with a sensitivity of 82% and a specificity of 81%. Furthermore, we tested the accuracy for the right parietal region separately for the delta power and peak frequency since these characteristics in this region have previously been identified to index the transition from MCI to dementia (Fernández et al., 2013). This resulted in an accuracy of 72% (sensitivity 70%; specificity 73%; area-under-the-curve 0.727; data not shown) for the superior part and an accuracy of 72% (sensitivity 63%; specificity 81%; area-under-the-curve 0.726; data not shown) for the inferior part of the right parietal cortex in the delta power, which is substantially higher than the values obtained for the global delta power (accuracy 66%; sensitivity 85%; specificity 46%; area-under-the-curve 0.698; **Figure 5A**). The accuracy for the peak frequency did not improve (56%; data not shown) when only the right parietal region was taken into account.

### Correlation with Cognition

**FIGURE 6:**
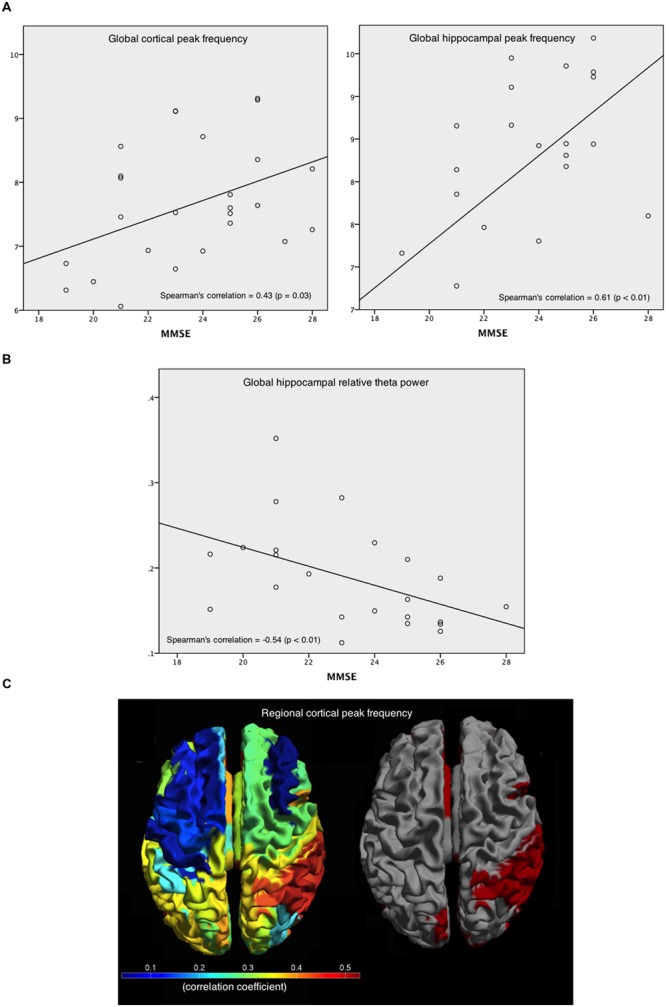
**Correlations between peak frequency and relative theta power in the patients’ cortical and hippocampal areas with MMSE. (A)** Spearman’s correlation coefficient between MMSE and peak frequency, averaged over all cortical areas (left), and over hippocampal areas (right). **(B)** Spearman’s correlation coefficient between MMSE and the relative theta power averaged over hippocampal ROIs. **(C)** Spearman’s correlation coefficients between MMSE and peak frequency for every cortical ROI (left), and the significant (red) and non-significant (gray) correlations (right).

Within AD patients and averaged over all cortical regions, the relative power values in the various frequency bands did not have a significant correlation with patients’ MMSE score, whereas the peak frequency showed a positive correlation [*r*(25) = 0.43; *p* < 0.05; **Figure 6A**, left]. For the hippocampus, a positive correlation with MMSE [*r*(25) = 0.61; *p* < 0.01; **Figure 6A**, right] was found. In addition, a negative correlation [*r*(25) = -0.54; *p* < 0.01] between relative theta power in the hippocampus and MMSE was found (**Figure 6B**). Regional correlations with peak frequency and MMSE score are shown in **Figure 6C** (left) while **Figure 6C** (right) shows the significant correlation values in red. Significant correlations between peak frequency and MMSE were mainly found for right parietal areas.

## Discussion

Using a novel non-invasive approach, we were able to localize oscillatory brain activity in cortical regions and the hippocampi of AD patients and controls. We found slower resting-state activity in AD patients compared to controls for the cortical regions as well as the hippocampi. In AD, the reduction of the peak frequency was widely present across the cortex with the exception of primary cortical areas, while theta power was increased throughout the entire cortex. The reduction in peak frequency, as well as increased relative theta power, was also observed throughout the hippocampus. The hippocampal peak frequency showed a strong positive correlation with cognition, with a corresponding negative correlation between cognition and hippocampal theta power. Cortical theta power was shown to be the best diagnostic discriminator.

### Hippocampal Oscillatory Activity

Here, we report for the first time results of virtual electrodes in patients with AD. These virtual electrodes were manually placed in a major disease hallmark region: the hippocampus, enabling the non-invasive study of hippocampal activity. We found a reduced peak frequency in the hippocampus of AD patients compared to controls indicative of slowing of brain activity in the hippocampus. Occipital regions are the main cortical generators of the dominant oscillatory activity in the alpha frequency range (8–13 Hz) during eyes-closed resting-state conditions (Klimesch, 1999). Therefore, the slowing of oscillatory brain activity is sometimes only considered using posterio-occipital MEG/EEG channels. However, some studies have shown that slowing also occurs in temporal and pre-frontal regions (Fernández et al., 2002; Criado et al., 2007). However, a general slowing of activity in cortical areas has been reported numerous times in both EEG (for a review see Jeong, 2004) and MEG data (e.g., de Haan et al., 2008). Our study adds to the current knowledge that in the early onset variant of the disease, where the hippocampal area is relatively spared compared to the late-onset variant of AD, slowing of oscillatory activity can already be observed in the hippocampus.

### Hippocampal Subdivision

Virtual electrodes were manually placed in six hippocampal regions (left and right anterior, central and posterior hippocampus). Previous research has suggested that anterior and posterior hippocampal areas have distinct specialization (Poppenk et al., 2013). This anterior–posterior hippocampal segmentation has also been found in a data-driven approach using diffusion-weighted images (Adnan et al., 2015). Hippocampal studies in animals showed not only distinct anatomical connections, but also functional dissimilarities between the ventral hippocampus (homolog of anterior hippocampus in humans) and the dorsal hippocampus (homolog of posterior hippocampus in humans; Fanselow and Dong, 2010), as Moser and Moser (1998) already suggested in their review in 1998. This so-called Moser-theory states that the anterior hippocampus modulates emotional and affective processes whereas the posterior hippocampus is specifically involved in memory function (Fanselow and Dong, 2010). In this view, it could be expected that AD patients would show more severe abnormalities in the posterior part of the hippocampus since memory processes are generally severely affected in these patients. Our finding showed a slowing of oscillatory brain activity in the hippocampi of AD patients regardless of the region. Therefore, we conclude that hippocampal dysfunction in terms of slowing of oscillatory activity is unlikely to be region dependent. An alternative explanation is that our non-invasive approach lacks the spatial resolution to detect subtle differences between the hippocampal sub-regions (see limitations below). Future studies should elucidate whether the functional connectivity profiles for these sub-regions differ, in line with the previously observed distinct anatomical connectivity profiles.

### Cortical Power

For the cortical regions, we found a lower peak frequency in AD patients in the association areas, whereas the primary cortical areas, such as primary visual cortex and sensorimotor cortices, were spared (**Figure 3C**). This finding is in line with the results of by Buckner et al. (2005), who described a hypothetical relationship across molecular, structural, and functional measures that all markedly overlap in association areas. But the primary cortices were spared. Our finding of lower peak frequencies in the association areas, a possible indication of an underlying pathological process in these areas, fits this. In our early onset AD patients, the peak frequency in occipital areas was not significantly lower compared to controls, whereas previous MEG studies found a reduced peak frequency when only assessing occipital- or occipitoparietal channels (Berendse et al., 2000; Montez et al., 2009). This contradiction might be a result of technical differences between the studies (signal space versus source space) or the use of an early onset AD group, which differs from late-onset in terms of atrophy [i.e., disproportionate atrophy in the posterior part of the brain while the hippocampi are relatively spared (Karas et al., 2007)]. The alpha rhythm is most dominant in the occipital regions of the brain. Unsurprisingly, the regions where in our study the relative power in the lower alpha was reduced were located in the occipital lobe. Furthermore, we found a widespread increase of relative theta power in patients with AD. The increase of theta power is in accordance with the general notion of slower brain activity in AD patients as well is the decrease of lower alpha power in the occipital area. The increase in low frequencies and decrease in high frequency bands in MEG in patients with AD has been published before (e.g., Berendse et al., 2000; Fernández et al., 2002, 2003, 2005, 2006a,b, 2013; Osipova et al., 2005; Poza et al., 2007, 2008; de Haan et al., 2008; Montez et al., 2009; Besga et al., 2010; Ranasinghe et al., 2014) and our regionally specific source-space approach is in line with these results.

### Correlation with Cognition

We found a strong correlation between MMSE scores and hippocampal peak frequency within the AD group. This correlation was stronger than the correlation found between MMSE score and cortical peak frequency as well as cortical/hippocampal theta power. Correlations between MMSE scores and MEG power in different frequency bands have been reported before, power in lower frequency bands were found to correlate negatively (Poza et al., 2008) and power in higher frequency bands to correlate positively (de Haan et al., 2008; Poza et al., 2008). Regionally, we found significant correlations in mainly right parietal areas between peak frequency and MMSE score whereas the temporal region did not show such correlations. Importantly, the absence of significant correlations between cognition and peak frequency in the neocortical temporal areas indicates that the observed relation between hippocampal peak frequency and cognition is unlikely to be due to signal leakage from nearby superficial cortical regions.

### Early Onset AD

The correlations between slowing of rhythmic activity and cognition, together with the diagnostic accuracy findings reported in this study, may reveal a functional dysfunction of the hippocampi in early onset AD. The cortical changes in AD patients are most outspoken, not only in terms of oscillatory activity, as suggested by the ROC curves in present study, but also in terms of atrophy (Karas et al., 2007). The hippocampi in early onset AD are relatively spared in terms of atrophy and pathological processes, while the posterior regions are relatively more affected as compared to the late-onset counterpart of the disease (Karas et al., 2007; Ossenkoppele et al., 2012). Therefore, early onset AD is usually accompanied by relatively spared scores on memory tests. For future research, it would be interesting to relate hippocampus atrophy scores to physiological data in this patient group. Interestingly, while the cortical theta power was the best discriminator between AD and controls, this parameter did not correlate with cognition. In contrast, the hippocampal peak frequency and relative theta power did not show good discriminative abilities, whereas significant correlations with cognition were found. The young AD group used in this study might explain this finding. In young patients with AD, typical findings like primary memory impairments are infrequently found. Instead of memory impairment and the accompanied hippocampal atophy, impairments in other cognitive domains, often accompanied by parietal atrophy, are more outspoken. Therefore, slowing in the posterior regions is likely to be a better diagnostic feature in this group than hippocampal slowing. When the disease severity increases, hippocampal atrophy and memory problems may arise. In this study, we found indications that hippocampal oscillatory activity is correlated to those features and may therefore indicate that despite the young age of the patients, the disease has already progressed to a more advanced disease stage.

### Methodological Considerations

Several potential limitations of this study should be taken into account. The estimated power spectra may have been influenced by methodological choices, such as the selection of artifact-free epochs. However, an independent researcher (IN, in acknowledgments) checked the selected epochs for quality and signs of drowsiness. We therefore expect that the epochs we have selected for our final analyses are artifact-free.

Magnetoencephalography has a lower spatial resolution for deeper regions (Hillebrand and Barnes, 2002). The spatial resolution of beamformer-reconstructed images of neuronal activity is inhomogeneous across the brain and typically ranges between 1 and 20 mm (Hillebrand et al., 2005), but for cortical regions it may be of the order of a few millimeters (Barnes et al., 2004). With regard to the placement of virtual electrodes in the hippocampus, we had to take this reduced spatial resolution into account. The hippocampus is approximately 7 cm in length, which makes a spatial resolution of 1–20 mm more than sufficient to detect anterior/posterior changes, although admittedly the exact spatial resolution for the hippocampal area is as yet unknown. Although it has been debated whether deeper brain regions can be detected by MEG, previous empirical data have shown that MEG beamformer techniques are able to reconstruct these regions, particularly when group averages are constructed (Quraan et al., 2011).

In this study, we used a single sphere as a model for the volume conductor. The accuracy of the reconstructed time-series may be improved in future studies by using more realistic volume conductor models (Lalancette et al., 2011), albeit at the expense of computational complexity and more elaborate preprocessing.

From a clinical perspective, the modest sample size, comorbidity and disease heterogeneity might be limitations for the present study. However, all patients had pathological biomarkers suggestive for AD (either obtained by CSF analysis or by PET scanning). The healthy control participants were not tested in a clinical setting, and therefore, clinical information about disease history (besides dementia or other neurological diseases) were not available. Therefore, confounding effects of concomitant illnesses could not be assessed. Also, the healthy control participants did not have known amyloid status so it is possible that amyloid pathology was present in some of the participants in this group. We therefore believe that the findings of this study are potentially an underestimation of the real group effects.

## Conclusion and Future Directions

In this resting-state MEG study, we were able to detect hippocampal activity in AD using beamformer-based virtual electrodes. We found slowing of oscillatory brain activity in cortical areas, as well as in the hippocampus, which correlated with general cognitive decline. The cortical association areas and the hippocampus are most prone to AD-related slowing. We postulate that this novel approach using virtual electrodes can be used in future research to quantify functional interactions between the hippocampi and cortical areas. This may give insight into the connectional disruptions that occurs in AD, not only within the cortex, but also between cortex and hippocampi, and even within the hippocampi.

## Author Contributions

ME made substantial contributions to the acquisition of data and analysis and drafted the manuscript. AH participated in the design of the study and revised the manuscript for important intellectual content, made substantial contributions to the interpretation of the data and provided technical support during the preprocessing of the data. WF participated in the design and coordination of the study and revised the manuscript for important intellectual content. CS participated in the design of the study and revised the manuscript for important intellectual content and made substantial contributions to the interpretation of the data. PS participated in the design of the study and revised the manuscript for important intellectual content. ES participated in the design of the study and revised the manuscript for important intellectual content and made substantial contributions to the interpretation of the data. All authors read and approved the final manuscript.

## Conflict of Interest Statement

The authors declare that the research was conducted in the absence of any commercial or financial relationships that could be construed as a potential conflict of interest.

## Footnotes

1 http://home.kpn.nl/stam7883/brainwave.html
