# Supplementary material for: Slowing of Hippocampal Activity Correlates with Cognitive Decline in Early Onset Alzheimer’s Disease. An MEG Study with Virtual Electrodes
Source: Front Hum Neurosci. 2016 May 20;10:238. doi: 10.3389/fnhum.2016.00238 (PMC4873509; doi:10.3389/fnhum.2016.00238)
Supplement: Supplementary file 1 [file Table_1.DOCX]

Table S1. The nomenclature for the different areas, including the corresponding number, based on the AAL atlas (automated anatomical labeling) as reordered by Gong et al. 2009.

| **ROI number in Gong atlas** | **Hemisphere** | **Cortical regions** | **Abbreviations** | **ROI number in Gong atlas** | **Hemisphere** | **Cortical regions** | **Abbreviations** |
| --- | --- | --- | --- | --- | --- | --- | --- |
| 1 | Left | Gyrus Rectus | REC | 40 | Right | Gyrus Rectus | REC |
| 2 | Left | Olfactory Cortex | OLF | 41 | Right | Olfactory Cortex | OLF |
| 3 | Left | Superior frontal gyrus, orbital part | ORBsup | 42 | Right | Superior frontal gyrus, orbital part | ORBsup |
| 4 | Left | Superior frontal gyrus, medial orbital | ORBsupmed | 43 | Right | Superior frontal gyrus, medial orbital | ORBsupmed |
| 5 | Left | Middle frontal gyrus orbital part | ORBmid | 44 | Right | Middle frontal gyrus orbital part | ORBmid |
| 6 | Left | Inferior frontal gyrus, orbital part | ORBinf | 45 | Right | Inferior frontal gyrus, orbital part | ORBinf |
| 7 | Left | Superior frontal gyrus, dorsolateral | SFGdor | 46 | Right | Superior frontal gyrus, dorsolateral | SFGdor |
| 8 | Left | Middle frontal gyrus | MFG | 47 | Right | Middle frontal gyrus | MFG |
| 9 | Left | Inferior frontal gyrus, opercular part | IFGoperc | 48 | Right | Inferior frontal gyrus, opercular part | IFGoperc |
| 10 | Left | Inferior frontal gyrus, triangular part | IFGtriang | 49 | Right | Inferior frontal gyrus, triangular part | IFGtriang |
| 11 | Left | Superior frontal gyrus, medial | SFGmed | 50 | Right | Superior frontal gyrus, medial | SFGmed |
| 12 | Left | Supplementary motor area | SMA | 51 | Right | Supplementary motor area | SMA |
| 13 | Left | Paracentral lobule | PCL | 52 | Right | Paracentral lobule | PCL |
| 14 | Left | Precentral gyrus | PreCG | 53 | Right | Precentral gyrus | PreCG |
| 15 | Left | Rolandic operculum | ROL | 54 | Right | Rolandic operculum | ROL |
| 16 | Left | Postcentral gyrus | PoCG | 55 | Right | Postcentral gyrus | PoCG |
| 17 | Left | Superior parietal gyrus | SPG | 56 | Right | Superior parietal gyrus | SPG |
| 18 | Left | Inferior parietal, but supramarginal and angular gyri | IPL | 57 | Right | Inferior parietal, but supramarginal and angular gyri | IPL |
| 19 | Left | Supramarginal gyrus | SMG | 58 | Right | Supramarginal gyrus | SMG |
| 20 | Left | Angular gyrus | ANG | 59 | Right | Angular gyrus | ANG |
| 21 | Left | Precuneus | PCUN | 60 | Right | Precuneus | PCUN |
| 22 | Left | Superior occipital gyrus | SOG | 61 | Right | Superior occipital gyrus | SOG |
| 23 | Left | Middle occipital gyrus | MOG | 62 | Right | Middle occipital gyrus | MOG |
| 24 | Left | Inferior occipital gyrus | IOG | 63 | Right | Inferior occipital gyrus | IOG |
| 25 | Left | Calcarine fissure and surrounding cortex | CAL | 64 | Right | Calcarine fissure and surrounding cortex | CAL |
| 26 | Left | Cuneus | CUN | 65 | Right | Cuneus | CUN |
| 27 | Left | Lingual gyrus | LING | 66 | Right | Lingual gyrus | LING |
| 28 | Left | Fusiform gyrus | FFG | 67 | Right | Fusiform gyrus | FFG |
| 29 | Left | Heschl gyrus | HES | 68 | Right | Heschl gyrus | HES |
| 30 | Left | Superior temporal gyrus | STG | 69 | Right | Superior temporal gyrus | STG |
| 31 | Left | Middle temporal gyrus | MTG | 70 | Right | Middle temporal gyrus | MTG |
| 32 | Left | Inferior temporal gyrus | ITG | 71 | Right | Inferior temporal gyrus | ITG |
| 33 | Left | Temporal pole: superior temporal gyrus | TPOsup | 72 | Right | Temporal pole: superior temporal gyrus | TPOsup |
| 34 | Left | Temporal pole: middle temporal gyrus | TPOmid | 73 | Right | Temporal pole: middle temporal gyrus | TPOmid |
| 35 | Left | Parahippocampal gyrus | PHG | 74 | Right | Parahippocampal gyrus | PHG |
| 36 | Left | Anterior cingulate and paracingulate gyri | ACG | 75 | Right | Anterior cingulate and paracingulate gyri | ACG |
| 37 | Left | Median cingulate and paracingulate gyri | DCG | 76 | Right | Median cingulate and paracingulate gyri | DCG |
| 38 | Left | Posterior cingulate gyrus | PCG | 77 | Right | Posterior cingulate gyrus | PCG |
| 39 | Left | Insula | INS | 78 | Right | Insula | INS |
