# Supplementary material for: Slowing of Hippocampal Activity Correlates with Cognitive Decline in Early Onset Alzheimer’s Disease. An MEG Study with Virtual Electrodes
Source: Front Hum Neurosci. 2016 May 20;10:238. doi: 10.3389/fnhum.2016.00238 (PMC4873509; doi:10.3389/fnhum.2016.00238)
Supplement: Supplementary file 2 [file Table_2.DOCX]

Table S2. The nomenclature for the areas, including the corresponding number, based on automated anatomical labeling (AAL) as reordered by Gong et al., (2009) that have significantly different (*p*<0.05, corrected) peak frequency values between AD patients and healthy controls after permutation testing. AD = Alzheimer’s disease; PF = peak frequency; SD = standard deviation.

| **ROI number in Gong atlas** | **Hemisphere** | **ROI name (abbriviations) in Gong atlas** | **PF value in AD patients (mean (SD))** | **PF value in healthy controls (mean (SD))** |
| --- | --- | --- | --- | --- |
| 1 | Left | REC | 7.10 (1.05) | 7.93 (0.72) |
| 2 | Left | OLF | 7.09 (0.98) | 8.07 (0.86) |
| 5 | Left | ORBmid | 6.87 (0.84) | 7.65 (0.61) |
| 6 | Left | ORBinf | 7.19 (0.85) | 8.01 (0.74) |
| 9 | Left | IFGoperc | 7.12 (0.94) | 7.95 (0.76) |
| 10 | Left | IFGtriang | 6.81 (0.77) | 7.83 (0.77) |
| 15 | Left | ROL | 7.87 (1.33) | 8.94 (0.72) |
| 17 | Left | SPG | 7.70 (1.43) | 8.86 (0.80) |
| 19 | Left | SMG | 8.12 (1.36) | 9.17 (0.72) |
| 29 | Left | HES | 7.94 (1.28) | 8.96 (0.68) |
| 33 | Left | TPOsup | 7.41 (0.92) | 8.37 (0.68) |
| 36 | Left | ACG | 6.74 (.74) | 7.78 (0.78) |
| 40 | Right | REC | 7.13 (0.83) | 7.98 (0.81) |
| 41 | Right | OLF | 7.23 (0.87) | 8.03 (0.81) |
| 42 | Right | ORBsup | 7.07 (0.87) | 8.00 (0.84) |
| 45 | Right | ORBinf | 7.05 (0.94) | 7.92 (0.89) |
| 46 | Right | SFGdor | 6.53 (0.61) | 7.38 (0.89) |
| 50 | Right | SFGmed | 6.56 (0.63) | 7.28 (0.81) |
| 54 | Right | ROL | 7.67 (1.21) | 8.86 (0.98) |
| 56 | Right | SPG | 7.82 (1.50) | 8.95 (0.65) |
| 57 | Right | IPL | 7.73 (1.41) | 8.98 (0.80) |
| 58 | Right | SMG | 7.84 (1.44) | 9.08 (0.74) |
| 59 | Right | ANG | 7.86 (1.44) | 9.03 (0.78) |
| 68 | Right | HES | 7.93 (1.21) | 8.97 (0.85) |
| 72 | Right | TPOsup | 7.51 (1.10) | 8.39 (0.80) |
| 75 | Right | ACG | 6.75 (0.79) | 7.84 (0.83) |
| 78 | Right | INS | 7.70 (1.25) | 8.65 (1.02) |
