# Supplementary material for: Slowing of Hippocampal Activity Correlates with Cognitive Decline in Early Onset Alzheimer’s Disease. An MEG Study with Virtual Electrodes
Source: Front Hum Neurosci. 2016 May 20;10:238. doi: 10.3389/fnhum.2016.00238 (PMC4873509; doi:10.3389/fnhum.2016.00238)
Supplement: Supplementary file 3 [file Table_3.DOCX]

Table S3. The nomenclature for the areas, including the corresponding number, based on automated anatomical labeling (AAL) as reordered by Gong et al., (2009) that have significantly different (*p*<0.05, corrected) power in different frequency bands between AD patients and healthy controls after permutation testing. *all but these regions where significantly different between groups.

| **ROI number in Gong atlas** | **Hemisphere** | **ROI name (abbriviations) in Gong atlas** | **ROI number in Gong atlas** | **Hemisphere** | **ROI name (abbriviations) in Gong atlas** |
| --- | --- | --- | --- | --- | --- |
| **Delta band** |  |  | **Lower Alpha band (continuation)** | | |
| 1 | Left | SPG | 62 | Right | MOG |
| 59 | Right | ANG | 63 | Right | IOG |
| 61 | Right | SOG | 64 | Right | CAL |
| 62 | Right | MOG | 65 | Right | CUN |
| 63 | Right | IOG | 66 | Right | LING |
| 70 | Right | MTG | 67 | Right | FFG |
| **Theta band*** |  |  | **Beta band** |  |  |
| 24 | Left | IOG | 2 | Left | OLF |
| 28 | Left | FFG | 3 | Left | ORBsup |
| 31 | Left | MTG | 5 | Left | ORBmid |
| 32 | Left | ITG | 6 | Left | ORBinf |
| 34 | Left | TPOmid | 10 | Left | IFGtriang |
| 63 | Right | IOG | 11 | Left | SFGmed |
| **Lower Alpha band** | |  | 33 | Left | TPOsup |
| 21 | Left | PCUN | 35 | Left | PHG |
| 22 | Left | SOG | 36 | Left | ACG |
| 23 | Left | MOG | 40 | Right | REC |
| 24 | Left | IOG | 41 | Right | OLF |
| 25 | Left | CAL | 42 | Right | ORBsup |
| 26 | Left | CUN | 45 | Right | ORBinf |
| 27 | Left | LING | 50 | Right | SFGmed |
| 28 | Left | FFG | 62 | Right | MOG |
| 56 | Right | SPG | 70 | Right | MTG |
| 61 | Right | SOG | 73 | Right | TPOmid |
|  |  |  | 75 | Right | ACG |
